# Prevalence of Trachoma at Sub-District Level in Ethiopia: Determining When to Stop Mass Azithromycin Distribution

**DOI:** 10.1371/journal.pntd.0002732

**Published:** 2014-03-13

**Authors:** Jonathan D. King, Tesfaye Teferi, Elizabeth A. Cromwell, Mulat Zerihun, Jeremiah M. Ngondi, Mesele Damte, Frew Ayalew, Zerihun Tadesse, Teshome Gebre, Ayelign Mulualem, Alemu Karie, Berhanu Melak, Mitku Adugna, Demelash Gessesse, Abebe Worku, Tekola Endashaw, Fisseha Admassu Ayele, Nicole E. Stoller, Mary Rose A. King, Aryc W. Mosher, Tesfaye Gebregzabher, Geremew Haileysus, Peter Odermatt, Jürg Utzinger, Paul M. Emerson

**Affiliations:** 1 The Carter Center, Atlanta, Georgia, United States of America; 2 Department of Epidemiology and Public Health, Swiss Tropical and Public Health Institute, Basel, Switzerland; 3 University of Basel, Basel, Switzerland; 4 The Carter Center, Addis Ababa, Ethiopia; 5 Department of Public Health and Primary Care, Institute of Public Health, University of Cambridge, Cambridge, United Kingdom; 6 The Amhara National Regional State Health Bureau, Bahir Dar, Ethiopia; 7 Francis I. Proctor Foundation, University of California San Francisco, San Francisco, California, United States of America; University of California San Francisco, United States of America

## Abstract

**Background:**

To eliminate blinding trachoma, the World Health Organization emphasizes implementing the SAFE strategy, which includes annual mass drug administration (MDA) with azithromycin to the whole population of endemic districts. Prevalence surveys to assess impact at the district level are recommended after at least 3 years of intervention. The decision to stop MDA is based on a prevalence of trachomatous inflammation follicular (TF) among children aged 1–9 years below 5% at the sub-district level, as determined by an additional round of surveys limited within districts where TF prevalence is below 10%. We conducted impact surveys powered to estimate prevalence simultaneously at the sub-district and district in two zones of Amhara, Ethiopia to determine whether MDA could be stopped.

**Methodology:**

Seventy-two separate population-based, sub-district surveys were conducted in 25 districts. In each survey all residents from 10 randomly selected clusters were screened for clinical signs of trachoma. Data were weighted according to selection probabilities and adjusted for correlation due to clustering.

**Principal Findings:**

Overall, 89,735 residents were registered from 21,327 households of whom 72,452 people (80.7%) were examined. The prevalence of TF in children aged 1–9 years was below 5% in six sub-districts and two districts. Sub-district level prevalence of TF in children aged 1–9 years ranged from 0.9–76.9% and district-level from 0.9–67.0%. In only one district was the prevalence of trichiasis below 0.1%.

**Conclusions/Significance:**

The experience from these zones in Ethiopia demonstrates that impact assessments designed to give a prevalence estimate of TF at sub-district level are possible, although the scale of the work was challenging. Given the assessed district-level prevalence of TF, sub-district-level surveys would have been warranted in only five districts. Interpretation was not as simple as stopping MDA in sub-districts below 5% given programmatic challenges of exempting sub-districts from a highly regarded program and the proximity of hyper-endemic sub-districts.

## Author Summary

Trachoma, the leading cause of preventable blindness, is targeted for “elimination as a public health problem” by the year 2020. National programs are implementing the recommended strategy of surgery, antibiotics, facial cleanliness, and environmental improvements (SAFE) to meet this target. Many programs are currently facing the decision of when to scale down interventions, particularly mass drug administration (MDA) of azithromycin. We implemented large population-based surveys in two different zones of the Amhara National Regional State of Ethiopia. Rather than conducting an impact assessment first at the district level, followed by additional sub-district-level surveys, we took a novel approach to measure the prevalence of trachoma at sub-district level to be able to make an immediate decision of whether to stop MDA. Over 72,000 people in 714 communities in 72 sub-districts were examined for clinical signs of trachoma. We identified only six sub-districts that met criteria for being able to stop MDA. Our work demonstrates that determining the prevalence of trachoma at sub-district level is feasible but requires significant resources. In this hyper-endemic setting, sub-district-level surveys were not needed in the majority of districts. Overall, the clinical data suggest some decline in trachoma within these areas since the SAFE strategy was implemented.

## Introduction

Trachoma accounts for approximately 3% of global blindness and is targeted for “elimination as a public health problem” by the year 2020 [1], [2]. Trachoma is estimated to be endemic in 53 countries of which 35 have begun scaling-up or have fully implemented the recommended SAFE strategy for reaching the elimination targets [3]. SAFE is the acronym for an integrated package of interventions to treat, control, and ultimately prevent new cases of blinding trachoma through surgery (S), antibiotic distribution (A), facial cleanliness (F), and environmental improvements (E) [4]. The ultimate intervention goal (UIG) for trachoma control, defined by the World Health Organization (WHO) as “elimination as a public health problem”, is achieving a transmission target: reducing trachomatous inflammation follicular in children aged 1–9 years (TF_1–9_) to less than 5%, and a morbidity target: less than 1 case of trachomatous trichiasis (TT) per 1,000 people [5].

WHO recommends annual mass drug administration (MDA) with azithromycin or tetracycline ophthalmic ointment in areas where the district-level prevalence of TF_1–9_ is greater than 10% [6]. A major challenge faced by national programs currently implementing SAFE is determining when to stop MDA. According to guidelines issued after the 2^nd^ global scientific meeting (GSM) on trachoma in 2003, the impact of SAFE on the prevalence of trachoma should be assessed after at least 3 years of implementation and MDA stopped where the prevalence of TF_1–9_ is determined to be less than 5% in any community [6], [7].

Realizing that estimating trachoma prevalence in every community in sub-Saharan Africa places a heavy burden on resource-constrained programs, the 3^rd^ GSM on trachoma was convened by WHO in July 2010 with the purpose of reviewing and clarifying guidelines for the implementation of impact assessment surveys, including the administrative level at which impact must be measured to stop MDA. The new guidelines suggest using the district as the evaluation unit (EU) on which to estimate trachoma prevalence, followed by a decision to continue SAFE or estimate trachoma prevalence through additional surveys at the sub-district level to determine whether MDA could be stopped. The new guidelines also suggest that “outcome surveys can be used to pronounce achievement of UIG for TF, if the sample size is powered to calculate estimates at the sub-district level” [8].

The purpose of this study was to apply this latter statement from the 3^rd^ GSM in two different programmatic settings in the Amhara region of Ethiopia by conducting trachoma outcome surveys at the sub-district level to determine where MDA might be stopped. A secondary aim of the study was to compare current estimates to baseline prevalence in order to monitor impact of interventions.

## Methods

### Ethics Statement

The study protocols for surveys in both South Wollo and South Gondar were reviewed and approved by the ethical review committee of the Amhara Regional State Health Bureau. Additionally, the study activities were approved by Emory University Institutional Review Board under protocol 079-2006. Due to the high rate of illiteracy, verbal informed consent was obtained and recorded prior to data collection rather than written information and a signed statement. Consent for trachoma examination and household interview was obtained from heads of households, individuals, and parents of minors according to the principles of the declaration of Helsinki.

### Study Site and Time Frame

Widespread implementation of the full SAFE strategy to all endemic areas in Amhara was initiated in 2007, which included outreach camps to provide surgery in addition to the existing static service; annual MDA with antibiotics; promotion of facial cleanliness and hygiene in schools and communities by health extension workers; community-level promotion of latrine construction and use through the health extension workers and environmental health officers. Provision of improved water sources was simultaneously supported by the development sector.

Previous data indicate that 6–12 months since the last round of MDA is the ideal time to conduct impact surveys to assess trachoma [9]. The outcome survey in South Wollo was conducted in December 2010 in 13 *woredas* (districts) which had received a third consecutive round of MDA in April 2010. The remaining eight *woredas* in South Wollo had not yet received three rounds. In South Gondar, training and field collection activities were conducted in the rainy season from late June to early August 2011 covering all 12 *woredas* in the zone. All *woredas* in South Gondar had received at least five rounds of MDA, the last round of which took place in November 2010.

### Sampling Methodology and Sample Size

**Figure 1:**
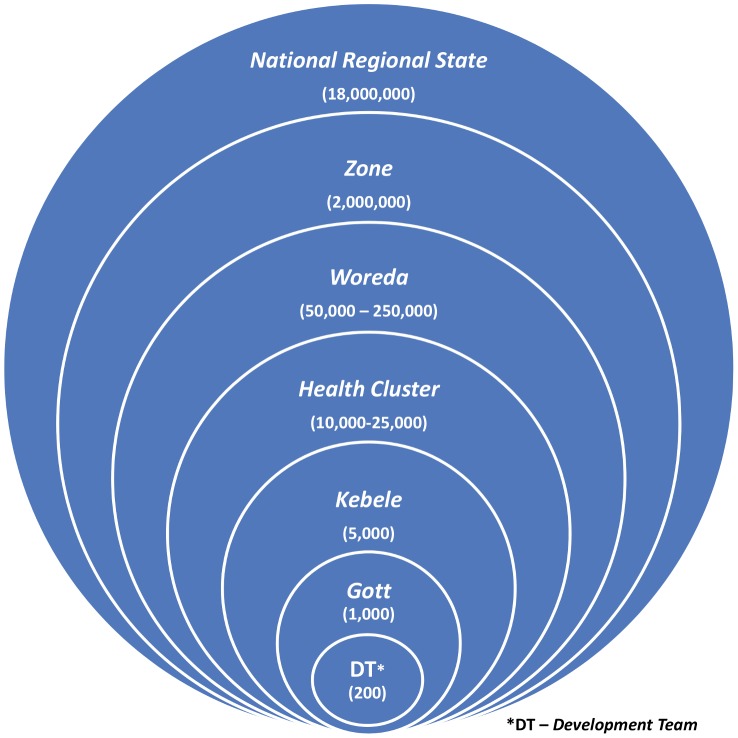
Administrative levels and respective population structure of Amhara National Regional State, Ethiopia.

The administrative levels of this area of Ethiopia and the respective population at each tier are presented in Figure 1 with the *woreda* being the district-level equivalent. From the 3^rd^ GSM report, a sub-district is defined as three or more villages with combined total population of at least 30,000 people [8]. Since no existing administrative level matched this description exactly, we joined geographically adjacent *health clusters* in groups to create a sub-district EU of approximately 50,000 cumulative population. *Gotts* are the smallest administrative unit for which there is population data available, which we used as our primary sampling unit or cluster. For each EU surveyed, *gotts* were selected from a line list arranged according to geographical distribution using a standard methodology [10]. *Gotts* are broken down into smaller administrative units called *development teams* (DT), which made ideal segments on which to base a modified segmentation design for equal probability sampling [10]. One DT was randomly selected in each *gott* and all persons present or absent residing in all households within the selected DT were registered and those present examined. Absent households were registered as non-consenting and were not replaced. Heads of household (or their adult representative) were interviewed in consenting households. Physical characteristics were recorded from direct observation.

To detect whether the prevalence of TF was 3% with 2% precision, we calculated a sample size of 558 children aged 1–9 years per sub-district EU. This was based on a 5% level of significance, design effect of 2, five persons per household with children aged 1–9 years composing 30% of the total population. To obtain the target sample size, population-based, cluster random sampling was utilized for both surveys. We surveyed 10 clusters per sub-district, including 40 households per cluster, which, based on our assumptions, would allow for an 8% non-response rate in children.

### Training and Quality Control

Prior to the survey data collection, teams participated in a 7-day, applied training and skills examination. Trachoma graders were responsible for diagnosing the clinical signs of trachoma using the simplified trachoma grading system [11]. Potential graders were trained to classify signs of trachoma using digital photographs, followed by examination with a standardized set of photographs where agreement was calculated for all signs. Poor performing participants were given additional instruction or assigned other duties. Remaining participants practiced examination and diagnosis of trachoma for at least 2 days in volunteer subjects of all ages resident in communities not selected for the survey. Finally, the participants took a reliability exam to measure their agreement with the “gold standard” grading of volunteer subjects. The “gold standard” was the consensus grade of the course trainers (ophthalmologist, trachoma program director, and trachoma survey consultant) experienced with the simplified trachoma grading system. Additionally, in South Wollo, digital photos were taken of each eye included in the inter-observer exam and reviewed to obtain consensus when trainers' grades were discordant. The agreement for all signs was generated, but selected graders were those achieving greater than 84% agreement and a Kappa ≥0.7 on grade TF.

Training for data recorders consisted of classroom instruction and field practice, which included testing and refining the data collection tools, applying the sampling strategy in the field, and taking geographical coordinates of survey households. Persons trained to serve as data recorders were also evaluated to assess their ability to follow protocol, perform the interview, and record responses correctly. The top performers were selected to work with selected graders to form the final survey teams. A total of 21 teams were deployed in South Wollo and 13 teams in South Gondar.

### Data Collection, Management, and Analysis

Each head of household was interviewed to assess demographics of the households and uptake of the SAFE strategy. All household residents were examined for the presence or absence of all five clinical signs of the simplified trachoma grading system in both eyes using a 2.5× binocular loupe and adequate light [11]. Additionally, in 99 *gotts* of South Gondar, DNA specimens of the tarsal conjunctivae of one randomly selected child aged 1–5 years per household were collected using ocular swabs for detection of infection with *Chlamydia trachomatis* by polymerase chain reaction (PCR) (at the time of writing this article, results were not yet available). The surveys also included non-trachoma indicators to measure outcomes of integrated program interventions for malaria in South Wollo and intestinal parasites in South Gondar. These data have been presented elsewhere [12].

Data were collected by recorders on paper survey forms in South Wollo zone. Forms were reviewed for completion by a team leader prior to leaving the *gott*. Data in South Gondar were collected electronically using *Swift Insights*, an Android (Google Inc.) application, operated on tablet computers [13]. In South Gondar, data recorders reviewed survey data after completing each household prior to initiating the next house. In both zones, supervisors met with each team at least once per two clusters surveyed to provide needed materials and collect completed forms. Data collected electronically were copied to an external micro SD card and downloaded every 2–3 days by the team's supervisor.

Paper-based survey data were double-entered in Microsoft Access by separate entry clerks, compared for discordance, and corrected with the original hard-copy. Electronic data were downloaded at the end of the survey separately for each team and converted from html to csv format. Data were analyzed using STATA version 13 (STATA Corp., College Station, United States of America). The inverse of the selection probabilities was calculated and used to weight the data in the analysis. Additionally, the analysis accounted for clustering at the *gott*, DT, and household levels by identifying the complex survey design and generating a robust estimate of variance [14]. Finite population corrections were provided for each stage of sampling. Sub-district-level data were aggregated to generate trachoma prevalence estimates at the district and zonal levels. Current prevalence estimates were compared with a regional baseline survey that provided zonal-level trachoma prevalence in 2006 [15] and, in South Gondar, from cross-sectional trachoma surveys prior to pilot interventions. The statistical significance of differences was assessed with a chi-square (Χ^2^) statistic accounting for the survey designs.

## Results

**Figure 2:**
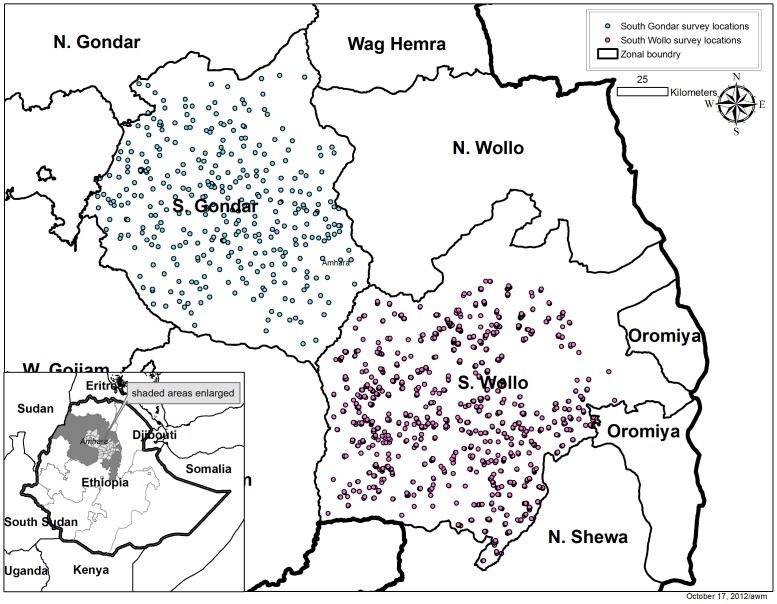
Surveyed communities in South Gondar and South Wollo zones of Amhara National Regional State, Ethiopia.

Teams successfully surveyed 714 of 720 communities from 72 EUs across the 25 *woreda*s in the two zones. The six *gotts* not surveyed were all in South Gondar and inaccessible during the rainy season. Figure 2 shows the geographical distribution of the 714 communities in the two zones of Amhara National Regional State, Ethiopia. A total of 89,735 residents were registered from 21,327 households of whom 72,452 (80.7%) were examined for clinical signs of trachoma. A summary description of the sample stratified by zone is shown in Table 1. Less than 2% of households encountered in selected *gotts* refused to participate in the survey. A higher proportion of residents were not available for examination (absent from the house) in South Gondar than in South Wollo (Χ^2^ = 1.73, p<0.001). The proportion of enumerated residents examined was lowest among the adult male population (data not shown). Among the target population, children aged 1–9 years, 95.8% and 94.9% of the enumerated children in South Wollo and South Gondar were examined, respectively. The proportion of these children with a clean face was 75.5% (95% confidence interval (CI) 72.8–78.0%; range by *woreda* 54.0–95.9%). The mean number of children in the 1–9 year age group sampled in each EU was 269.7 (standard deviation (SD) 50.6). The mean number of people examined of all ages in each EU in South Wollo was 938.9 (SD 192.0). The prevalence estimates at the EU were based on a mean sample size of 487.0 (SD 45.8) children 1–9 years of age and 1,073.7 (SD 106.8) total individuals examined per EU.

**Table 1:** Sample description in South Wollo and South Gondar by *woreda* (district) and sub-district evaluation unit (EU).

*Woreda*	Total EU*	Total *gotts*	Houses		Individuals		Proportion male		Adults≥15 years		Children 1–9 years	
			surveyed	%**	examined	%**	examined	enumerated	examined	%**	examined	%**
Adjibar Saiynt	3	30	793	100	1,113	98.7	40.4	44.9	1,375	79.8	747	97.8
Albuko	2	20	440	89.2	1,504	69.7	46.6	51.9	894	69.2	429	79.7
Borena	3	30	895	98.7	3,360	90.6	46.3	48.6	1,829	94.7	971	99.7
Jamma	3	30	893	99.2	3,622	95.7	48.5	49.3	2,152	95.2	965	98.1
Kelala	3	30	719	92.7	2,415	77.8	44.4	48.8	1,342	75.9	711	88.0
Kutaber	2	20	477	96.4	1,798	87.6	46.8	49.7	1,036	85.0	451	94.6
Legambo	4	40	859	99.0	3,279	86.4	46.8	50.2	1,832	82.2	952	97.3
Legahida	2	20	421	100	1,725	96.6	49.3	50.3	962	95.3	493	98.4
Mehal Saiynt	2	20	506	100	1,942	87.4	45.6	48.7	1,021	82.6	622	98.1
Mekidela	3	30	991	99.8	3,819	98.6	47.4	47.4	2,285	99.1	1056	99.3
Tenta	3	30	751	99.7	2,349	74.0	43.1	49.9	1,368	69.7	657	92.9
Wogedi	3	30	792	99.9	2,714	86.5	43.3	46.5	1,472	83.0	841	96.3
Woreillu	3	30	726	99.3	2,903	89.7	45.3	48.0	1,640	86.3	813	98.2
**South Wollo**	36	360	9,263	98.2	33,800	87.0	45.8	48.6	19,208	84.9	9,708	95.8
Debre Tabor	1	10	368	100	1,109	69.7	36.7	44.4	515	55.8	519	96.1
Dera	4	39	1,325	99.9	4,350	78.0	41.6	48.2	1,903	65.8	2,128	96.4
East Estie	4	35	1,186	99.7	3,861	74.1	41.6	48.3	1,776	62.3	1,828	96.5
Ebinat	4	40	1,309	100	4,256	79.1	41.3	47.8	1,915	67.1	2,036	97.5
Farta	4	40	1,269	100	4,248	75.5	43.3	49.3	1,984	64.9	1,907	96.2
Fogera	3	30	985	98.6	3,415	77.2	43.3	49.1	1,607	69.4	1,399	92.9
Lay gayint	4	40	1,457	99.3	4,497	75.1	39.5	46.4	2,193	66.1	1,971	94.0
Libokem	3	30	1,020	99.3	3,289	74.3	41.0	48.3	1,529	65.8	1,482	92.9
Simada	4	40	1,407	99.5	4,135	73.7	38.8	46.1	2,011	65.5	1,800	91.0
Tach gayint	2	20	690	99.9	2,185	79.4	39.3	46.3	1,092	71.7	937	93.8
West Estie	2	20	689	100	2,180	77.6	42.4	47.7	1,033	67.6	1,011	96.0
Woreta town	1	10	359	100	1,127	75.5	39.3	46.7	509	62.5	543	96.8
**South Gondar**	36	354	12,064	99.6	38,652	76.0	41.0	47.6	18,067	65.8	17,561	94.9

*Sub-district evaluation unit (EU).

** excluding 1.8% and 0.4% of households in South Wollo and South Gondar for which consent was not given.

**Figure 3:**
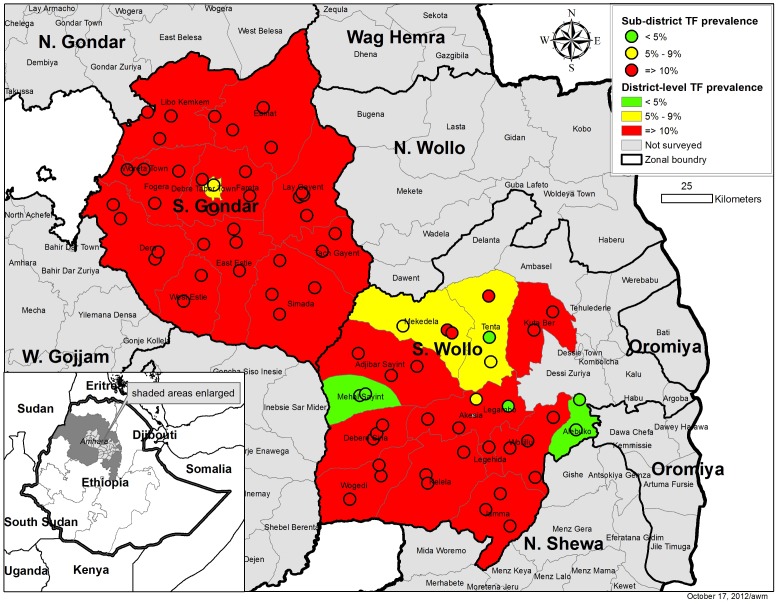
Distribution of trachomatous inflammation folliclar (TF) among children aged 1–9 years in Amhara National Regional State, Ethiopia. Data are stratified by *woreda* and evaluation unit.

The sub-district-level and *woreda*-level prevalence of TF_1–9_, classified as <5%, 5–9%, and ≥10% is shown for both zones in Figure 3. Overall, the prevalence of TF_1–9_ was below 5% in six EUs and between 5% and 9% in an additional three EUs in South Wollo (Table 2). According to the proposed WHO guidelines, where the *woreda*-level TF_1–9_ prevalence is <10%, MDA with azithromycin may be stopped in EUs where TF_1–9_ prevalence is less than 5%. In two *woredas* (Albuko and Mehal Saiynt; see Table S1), at both the EU and *woreda* levels, the prevalence of TF_1–9_ was below the 5% threshold and warranted stopping MDA in the entire *woreda*. One additional EU in Tenta *woreda* also warranted stopping MDA under the guidelines. The additional EU with TF_1–9_ prevalence below 5% in Legambo *woreda*, while not identified in the suggested way of district then sub-district surveys, meets the UIG criteria. TF_1–9_ in 4 of 13 *woredas* was <10% and would have warranted further sub-district-level surveys. Neither at the *woreda*-level nor in any single EU was the prevalence of TF_1–9_ below 5% among children in South Gondar (see Table S2). Targeted MDA at the sub-district level is recommended in three *woredas* (Tenta and Mekidela in South Wollo, and Debre Tabor in South Gondar) where TF_1–9_ prevalence was between 5% and 9%. For 20 of 25 *woredas* across the two zones, WHO guidelines suggest there was no need for sub-district level assessment and MDA with azithromycin should continue for at least an additional 3 years.

**Table 2:** Summary of survey findings and recommended programmatic strategy for trachoma elimination in South Wollo and South Gondar Zone, Ethiopia.

	South Wollo	South Gondar
**EU** * **with TF<5%**	6/36	0/36
**EU** * **with 5%<TF<10%**	3/36	1/36
***woreda*** ** TF<5%**	2/13	0/12
***woreda*** ** 5%<TF<10%**	2/13	1/12
***woreda*** ** TT<0.1%**	1/13	0/12
**Recommended program strategy** ‡	Stop MDA in 6/36 EU with TF<5%. Conduct targeted MDA in three EU with TF 5–9% (within 2 *woredas*). Continue district-wide MDA in nine *woredas* for 3 more years. Enhance provision of TT surgery in 12 *woredas*.	Conduct targeted MDA in one EU (within one *woreda*) with TF 5–9%. Continue district-wide MDA in 11 *woredas* for 3 more years. Enhance provision of TT surgery in 12 *woredas*.

*Sub-district evaluation unit (EU).

‡ Interpretation of WHO guidelines.

The prevalence of TF_1–9_ in the target age group for the combined area in South Wollo zone was 26.4% (95% CI 23.7–29.1%; range by *woreda* 0.9–67.0%; range by EU 0.9–76.9%) (see Table S1). Prevalence of trachomatous inflammation intense (TI) was 4.3% (95% CI 3.2–5.4%; range by *woreda* 0.7–22.0%; range by EU 0.0–28.6%). Among all residents, the prevalence of TT was 1.3% (95% CI 1.1–1.5%; range by *woreda* 0.0–3.1%; range by EU 0.0–4.3%). In South Gondar (see Table S2), TF_1–9_ prevalence was 25.9% (95% CI 23.8–27.9%; range by *woreda* 8.6–44.9%; range by EU 8.6–55.7%). Prevalence of TI was 7.0% (95% CI 6.2–7.8%; range by *woreda* 4.3–10.7%; range by EU 1.4–13.4%). The prevalence of TT was 1.8% (95%CI 1.6–2.0%; range by *woreda* 1.0–3.5%; range by EU 0.6–4.2%) among the total examined population.

**Figure 4:**
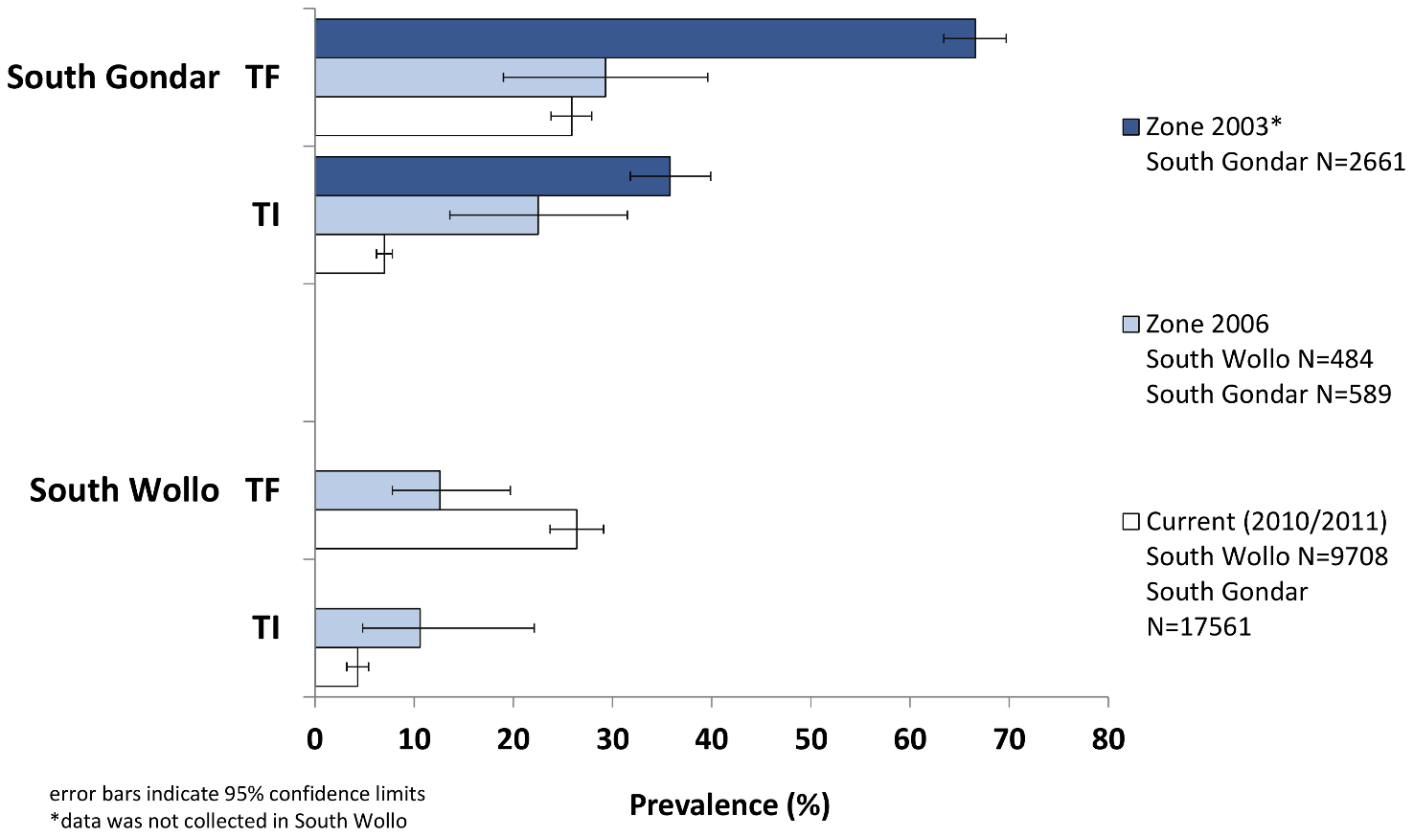
Prevalence of active trachoma and clinical signs among children aged 1–9 years in Amhara National Regional State, Ethiopia. Data are shown for the years 2003, 2006, and 2010–2011.

In South Wollo, the prevalence of TF_1–9_ for the combined 13 *woredas* was higher than the baseline zonal estimate in 2006 (p = 0.013, Figure 4). The prevalence of TI in the current survey was lower than the 10.6% estimate in 2006 (p = 0.053). Compared to estimates from two previous, cross-sectional, cluster randomized surveys in South Gondar, the prevalence of TF_1–9_ decreased from 2003 (p<0.001) but did not change significantly from the estimate in 2006 (p = 0.509). The current TI prevalence among children in South Gondar was lower than previous estimates, 35.8% in 2003 (p<0.001) and 23.3% in 2006 (p<0.001).

## Discussion

Recommendations of the 3^rd^ GSM report suggested that, after *woreda*-level surveys indicated a prevalence of TF_1–9_ below 10%, sub-district-level estimates were required before making an ultimate decision to cease MDA [8]. In each zone, we chose to implement surveys in a single exercise to estimate the prevalence of TF_1–9_ at the sub-district level in all districts eligible for outcome surveys to avoid an additional round of surveys. We chose this strategy for one main reason: to obtain a definite answer for all areas in the two zones within a timeframe feasible for mobilizing and implementing an annual MDA. Planning and implementing impact surveys are vital but compete for financial and human resources against the ongoing implementation of program interventions, which require decisions for planning well ahead of scheduled activities. For instance, submission of the application for donated azithromycin and planning of its distribution occurs 8–20 months in advance of the intended distribution. Instead of having a scenario where two surveys need to be completed prior to the forecasting of drug needs, we felt a single survey, powered to estimate the prevalence of both the sub-district and the district was preferred. However, in this trachoma hyper-endemic region of Ethiopia, conducting impact assessments first at the *woreda* level would have provided necessary data for making the decision to continue SAFE in 20 of the 25 *woredas* avoiding the need to survey as many communities as was assessed.

According to the latest WHO guidelines, trachoma control programs will eventually need to implement sub-district-level surveys. The experience from implementing these large-scale surveys in both zones, demonstrates that such surveys designed to simultaneously measure disease prevalence at the sub-district and district levels to evaluate impact of the SAFE strategy are indeed feasible. However, the process was neither without challenge nor the outcome without limitations. For instance, over 50 people were required to implement each survey, including data recorders, clinical examiners, drivers, supervisors, logisticians, and a coordinator. During the training in both zones, only about half of the clinical examiners met our pre-set criteria to participate in the study even after several days of applied training. The final number of survey teams is dependent on the number of examiners who can accurately apply the trachoma grading system. If fewer examiners meet the criteria, fewer teams can be deployed, which increases the number of clusters each team must cover and the total number of days in the field. In Amhara, the majority of *gotts* are accessible only on foot or horseback. Once in the community, teams walked long distances between houses within a selected DT to survey each household and often slept overnight in the community to repeat the process the next day. Accessibility in the field is further constrained when surveys are conducted in the rainy season; however, due to MDA forecasting needs and program implementation schedule, carrying out the survey in the rainy season was unavoidable in South Gondar. In South Wollo, around 12,000 paper-based surveys were distributed to survey teams for data collection. The printing, sorting, labeling, and distribution of these forms took 18 person-days. Double data entry, comparison, and correction took over 200 person-days and seven rental laptop computers. Electronic data collection in South Gondar streamlined data management and saved substantial time [13]. Overall, from planning to data analysis, each of these surveys took approximately 3 months to complete, including 30 days of field work. Our survey cost estimates are incomplete until the ocular swab specimens are processed, but a generalizable median cost per cluster of US$ 311 for conducting population-based prevalence surveys of trachoma clinical signs was estimated previously [16].

Our decision to test this novel approach was also influenced by an assumption that perhaps after 3–5 years of SAFE interventions, TF_1–9_ would be reduced to less than 10%. Implementation of the full SAFE strategy, including five consecutive, annual mass azithromycin distribution campaigns did not result in a reduction of TF_1–9_ prevalence below the UIG targets in any sub-district within the trachoma hyper-endemic setting of South Gondar. The impact on TF_1–9_ after 3 consecutive years of SAFE implementation was not as clear in South Wollo, where the overall prevalence of TF_1–9_ was higher than the zonal-level prevalence estimated prior to SAFE interventions. Yet, TF_1–9_ prevalence in a fraction of the sub-districts assessed and two of 13 *woredas* were below the 5% UIG. According to WHO guidelines these areas are eligible to stop MDA for trachoma. Our sample of 9,708 children aged 1–9 years across 360 communities in South Wollo provided much more accurate and precise estimates than at baseline surveys. Indeed, the baseline prevalence estimates for the entire zone of South Wollo were derived from 484 children across 16 communities [15]. The 12.6% TF_1–9_ estimate for South Wollo in 2006 was the lowest for all 10 zones in the region and the only zone estimated below 20% TF_1–9_ prevalence. In South Wollo, the observed difference in TF_1–9_ prevalence compared to the baseline survey was likely the results of an underestimate of the true TF_1–9_ prevalence at baseline due to chance. Four of the randomly selected communities in 2006 were from *woredas* where the current prevalence was lowest which might have contributed to a lower prevalence estimate if TF_1–9_ prevalence was also less common at baseline in these areas.

Additional prevalence data from South Gondar prior to 2006 suggest a decline of TF prevalence since the initiation of the program (Figure 2), but a reduction in TF_1–9_ from 2006 to 2011 was not observed in the recent survey. Reduction in TF_1–9_ below 5% has been documented in other programs after SAFE implementation, but in those settings the prevalence of TF_1–9_ at baseline was lower than in Amhara [17], [18]. TF_1–9_ prevalence in South Sudan (also hyper-endemic for trachoma) was reduced to below 5% after high community uptake of SAFE interventions but this was observed only in one of four intervention sites [19].

One conclusion that could be made from the results is that a lack of reduction in TF_1–9_ indicates that trachoma has not been controlled and continued MDA is needed. Alternatively, trachoma research studies from another region of Ethiopia have documented slow resolution of clinical signs after MDA has successfully reduced infection [20]. In one study, although a mean prevalence of 43.5% TF was measured among children post 3 years of azithromycin MDA, in a third of the participating communities, there was no evidence of *C. trachomatis* infection [21]. A separate study from the same area found infection prevalence of 2–3% in the context of 35% TF and/or TI prevalence after 3 years of either annual or biannual MDA of azithromycin [22]. These studies suggest difficulties in basing interpretations of impact and decisions to stop antibiotic distribution on TF prevalence alone. A reduction in the more severe active trachoma grade, TI, was observed in our surveys compared to baseline data in both zones. A reduction in TI was also documented in the first impact assessment conducted in five *woredas* after 3 years of SAFE implementation in Amhara [23]. An ordinal analysis of those data suggested a combination of TF and TI might serve as a better impact indicator than TF alone, which is also supported from a recent study in Tanzania [24], [25].

According to current WHO guidelines, all *woredas* of South Gondar warrant ongoing MDA with antibiotics [6]. After having received five to seven rounds of MDA, the prevalence of *C. trachomatis* infection is uncertain, but data from other studies in the Amhara region raise the question of whether additional rounds will provide further specific trachoma-related benefits [21]–[23]. In hyper-endemic communities from a different region where region-wide trachoma control interventions are not yet underway, a rebound of *C. trachomatis* infection was observed 6–24 months after no evidence of infection could be determined post-azithromycin distribution [26]. In the Amhara setting where there is region-wide intervention and fewer opportunities for exposure to re-infection, infection data will provide crucial, additional evidence for MDA decisions even though relative stopping thresholds based on infection have only been suggested from mathematical models [27]. We have not had the ability to process the collected conjunctival DNA swabs to date due to constraints in the local availability of reagents and national policies regarding exporting clinical specimens.

Although these surveys are a landmark for trachoma control, our results should be interpreted in the context of the following limitations. The actual sample size achieved at the sub-district level was lower than the estimated sample size desired. We assumed a household size of five persons based on our prior work in Ethiopia, when in fact the mean household size was smaller (i.e., four). The number of households in a DT was not 50 households as projected, but varied greatly within a district. In South Wollo, we found the DTs to be much smaller than anticipated. To correct for this observation in South Gondar, multiple DTs were randomly selected in each cluster. Yet, even in South Gondar the actual sample size did not meet expectations. Selecting different numbers of segments per cluster results in unequal probability sampling which had to be adjusted for by weighting the analysis according to the selection probabilities and doing so decreases the precision of the estimates. If the actual point prevalence findings for TF at the sub-district level had been near the stopping threshold, then this may have been a reason for concern; however, most of the sub-districts were well above 5% so this should not bias decisions regarding MDA implementation. Another limitation inherent with utilizing clinical signs of the simplified trachoma grading system is that diagnosis is subjective and has high variability between examiners of the same patient [28]. While we took every means possible to select the most capable, reliable graders, there will always be the chance of systematic misclassification. Taking photographs of the everted lid has been shown to provide an objective measure of findings in some studies, but not in all settings and not at the scale of the current study [29]–[31]. We used photographs only during the standardization of the graders but capable cameras were not deployed with any of the 34 survey teams. With the rapid development in technology of devices which are used for electronic data collection, it might be useful to explore improvements in the capability of the smart device to capture photos of the tarsal conjunctivae [32].

The decision to stop MDA is not straightforward. Strictly following WHO guidelines, Amhara Regional Health Bureau could cease MDA in six EUs in South Wollo. Programmatically, planning and coordination of interventions is done by the district and it is very difficult (politically and logistically) to exempt part of the district while the other part continues to receive a perceived benefit from the intervention. Scientifically, these six EUs are within *woredas* geographically surrounded by areas where the prevalence of TF warrants continued MDA (Figure 3). The spatial distribution of trachoma and correlation of trachoma between sub-districts may have implications on how stop-MDA decisions are made. Additionally, identification of factors at the community and geographical level associated with trachoma might assist in the prediction of the presence or absence of trachoma post-intervention and perhaps help minimize the amount of communities to be surveyed [33].

The total cumulative output of SAFE activities through December 2010 in Amhara, as reported by the Health Bureau include: 192,922 persons operated to correct trichiasis, 50.9 million doses of azithromycin distributed, 3,428 kebeles with ongoing trachoma-specific health education and over 1.8 million household latrines constructed (unpublished data). Facial cleanliness was specifically promoted in 1,324 schools. The proportion of children with clean faces had not changed since 2006 [15], indicating either that face washing is not being adopted or the measurement is confounded by other factors making this indicator difficult to interpret [34]. Field reports of antibiotic coverage ranged from 80–95% in each round, but these administrative reports were not validated [35]. In addition to clinical evaluation criteria on which to determine whether trachoma is no longer a public health problem, the compliance with the SAFE interventions, particularly hygiene and sanitation, should be required. Low prevalence of clinical trachoma signs in areas with low compliance of F and E interventions might be interpreted differently than areas where household latrine coverage, water access, and use for face washing is high. Such indicators have been measured in these zones and show increases in household sanitation and water access [14]. Additional investigations are warranted to determine whether high coverage of household-level access to water and sanitation supports sustained reduction of trachoma prevalence after MDA has stopped [36].

In conclusion, this study demonstrated that our methodology was capable of providing estimates at both the district and sub-district levels in a single survey, although minor adjustments would be needed to consistently meet the required sample size at the sub-district level. Implementing the survey at the sub-district level irrespective of the district-level TF prevalence might identify sub-districts which have met the control threshold for TF when the district level prevalence remains above 10%, but this was rare. Sub-district-level surveys would not have been warranted in most of the districts assessed, even after 5 years of SAFE interventions. This supports the current recommended approach of conducting impact assessments at the district level first in similar trachoma hyper-endemic settings. To obtain the sub-district-level estimates, 360 communities in a single zone had to be surveyed. While more feasible than surveying every village as suggested by the previous WHO recommendations, this number of clusters surveyed in each zone was greater than that surveyed in some national-level standardized surveys such as the multiple indicator cluster survey and malaria indicator survey. Realizing the logistical challenges posed by carrying out sub-district-level surveys and the difficulty of interpreting TF prevalence to make program decisions, begs the question: is there a better way to determine whether MDA could be stopped?

## Supporting Information

Checklist S1 **STROBE checklist.**
(DOC)

Table S1 Prevalence* of trachoma clinical signs by sub-district evaluation unit (EU) and ***woreda*** **in South Wollo, Ethiopia, 2010.**
(DOCX)

Table S2 Prevalence* of trachoma clinical signs by sub-district evaluation unit (EU) and ***woreda*** **in South Gondar, Ethiopia, 2011.**
(DOCX)
