# Supplementary material for: Prevalence of Trachoma at Sub-District Level in Ethiopia: Determining When to Stop Mass Azithromycin Distribution
Source: PLoS Negl Trop Dis. 2014 Mar 13;8(3):e2732. doi: 10.1371/journal.pntd.0002732 (PMC3953063; doi:10.1371/journal.pntd.0002732)
Supplement: Table S1 — Prevalence* of trachoma clinical signs by sub-district evaluation unit (EU) and woreda in South Wollo, Ethiopia, 2010. (DOCX) [file pntd.0002732.s002.docx]

**Table S1: Prevalence* of trachoma clinical signs by sub-district evaluation unit (EU) and *woreda* in South Wollo 2010**

| **South Wollo** | **EU** | **Children** | **TF** | | **TI** | | **All ages** | **TT** | |
| --- | --- | --- | --- | --- | --- | --- | --- | --- | --- |
| ***woredas*** |  | **1-9 years** | **%** | **95% CI** | **%** | **95% CI** |  | **%** | **95% CI** |
| **Adjibar Saiynt** | 1 | 284 | 27.2 | 10.9-53.1 | 2.2 | 0.7-6.5 | 1011 | 1.5 | 0.8-2.8 |
|  | 2 | 206 | 15.2 | 6.0-33.7 | 4.8 | 1.4-15.3 | 638 | 1.4 | 0.5-3.8 |
|  | 3 | 257 | 56.9 | 49.1-64.5 | 9.2 | 6.6-12.7 | 721 | 4.3 | 2.9-6.4 |
| woreda-level | | 747 | 30.7 | 20.7-42.9 | 4.6 | 2.9-7.2 | 2370 | 2.1 | 1.5-3.0 |
| **Albuko** | 1 | 203 | 1.0 | 0.2-5.2 | 4.8 | 1.8-12.5 | 769 | - | - |
|  | 2 | 226 | 0.9 | 0.2-3.4 | 3.6 | 1.4-9.0 | 735 | - | - |
| woreda-level | | 429 | 1.0 | 0.3-2.9 | 4.2 | 2.1-8.5 | 1504 | - | - |
| **Borena** | 1 | 284 | 15.7 | 11.2-21.7 | 2.3 | 0.6-9.3 | 1066 | 1.7 | 0.9-3.1 |
|  | 2 | 352 | 16.2 | 12.5-20.8 | 2.1 | 1.1-4.0 | 1191 | 1.2 | 0.7-2.1 |
|  | 3 | 335 | 12.8 | 6.9-22.5 | 0.5 | 0.1-3.2 | 1103 | 0.7 | 0.3-1.8 |
| woreda-level | | 971 | 15.1 | 12.2-18.5 | 1.7 | 0.9-3.3 | 3360 | 1.2 | 0.8-1.8 |
| **Jamma** | 1 | 303 | 18.5 | 0.3-36.7 | 3.8 | 1.2-11.7 | 1095 | 3.6 | 2.0-6.3 |
|  | 2 | 368 | 25.0 | 11.4-46.4 | 6.6 | 4.2-10.2 | 1388 | 0.3 | 0.1-1.2 |
|  | 3 | 294 | 23.3 | 17.3-30.6 | 6.1 | 4.1-9.0 | 1139 | 0.6 | 0.3-1.3 |
| woreda-level | | 965 | 21.8 | 13.0-34.3 | 5.3 | 3.3-8.4 | 3622 | 1.7 | 1.1-2.5 |
| **Kelala** | 1 | 214 | 19.6 | 13.9-27.0 | 1.8 | 0.6-5.8 | 778 | 2.8 | 2.2-3.5 |
|  | 2 | 260 | 26.4 | 15.4-41.5 | 3.8 | 1.4-9.9 | 876 | 1.5 | 0.9-2.6 |
|  | 3 | 237 | 36.7 | 26.1-48.8 | 2.4 | 0.6-9.1 | 761 | 2.3 | 1.4-3.5 |
| woreda-level | | 711 | 28.9 | 22.3-36.6 | 2.6 | 1.2-5.3 | 2415 | 2.3 | 1.8-2.9 |
| **Kutaber** | 1 | 190 | 20.8 | 11.7-34.2 | 7.7 | 2.2-23.6 | 821 | 1.7 | 0.5-5.9 |
|  | 2 | 261 | 22.4 | 13.7-34.3 | 0.7 | 0.2-3.1 | 977 | 0.5 | 0.2-0.9 |
| woreda-level | | 451 | 21.7 | 15.0-30.2 | 3.8 | 1.2-11.5 | 1798 | 1.1 | 0.4-2.8 |
| **Legahida** | 1 | 281 | 76.9 | 63.4-86.5 | 28.6 | 15.4-46.9 | 917 | 2.0 | 1.0-4.0 |
|  | 2 | 212 | 44.3 | 28.6-61.4 | 7.1 | 3.4-14.1 | 808 | 0.6 | 0.3-1.4 |
| woreda-level | | 493 | 67.0 | 55.3-76.9 | 22.0 | 12.6-35.6 | 1725 | 1.6 | 0.9-2.8 |
| **Legambo** | 1 | 243 | 4.2 | 1.8-9.6 | 0.0 | - | 889 | - | - |
|  | 2 | 217 | 7.5 | 4.5-12.1 | 0.0 | - | 723 | - | - |
|  | 3 | 259 | 38.4 | 29.1-48.6 | 2.6 | 1.5-4.5 | 887 | 0.7 | 0.2-1.8 |
|  | 4 | 233 | 24.4 | 19.7-29.7 | 1.8 | 0.5-6.0 | 780 | 0.5 | 0.2-1.2 |
| woreda-level | | 952 | 20.7 | 16.6-25.4 | 1.3 | 0.7-2.4 | 3279 | 0.3 | 0.2-0.6 |
| **Mehal Saiynt** | 1 | 313 | 1.1 | 0.2-5.1 | 2.4 | 0.8-6.7 | 1005 | 1.0 | 0.5-2.1 |
|  | 2 | 309 | 3.5 | 1.7-7.1 | 2.5 | 1.0-5.9 | 937 | 1.3 | 0.4-4.2 |
| woreda-level | | 622 | 2.2 | 1.0-4.5 | 2.4 | 1.2-4.8 | 1942 | 1.1 | 0.5-2.3 |
| **Mekidela** | 1 | 384 | 10.4 | 4.1-23.8 | 0.4 | 0.1-1.8 | 1429 | 0.2 | 0.1-0.6 |
|  | 2 | 319 | 6.9 | 3.5-13.7 | 1.2 | 0.5-3.0 | 1070 | 0.8 | 0.3-2.1 |
|  | 3 | 353 | 13.7 | 4.7-33.6 | 2.1 | 0.8-5.7 | 1320 | 0.1 | 0.0-0.6 |
| woreda-level | | 1056 | 9.5 | 5.6-15.5 | 1.2 | 0.7-2.3 | 3819 | 0.5 | 0.2-1.1 |
| **Tenta** | 1 | 219 | 15.1 | 9.3-23.7 | 1.9 | 0.6-6.6 | 776 | 2.5 | 1.2-5.1 |
|  | 2 | 222 | 3.6 | 1.7-7.5 | 0.4 | 0.1-2.5 | 833 | 0.7 | 0.3-1.8 |
|  | 3 | 216 | 7.8 | 3.8-15.5 | 0.0 | . | 740 | 0.3 | 0.1-1.5 |
| woreda-level | | 657 | 8.5 | 5.7-12.6 | 0.7 | 0.0-1.5 | 2349 | 1.1 | 0.6-1.8 |
| **Wogedi** | 1 | 313 | 30.8 | 19.8-44.6 | 7.2 | 2.4-19.6 | 1048 | 3.0 | 2.1-4.4 |
|  | 2 | 273 | 23.0 | 11.9-39.7 | 5.4 | 2.7-10.6 | 858 | 2.7 | 1.8-4.2 |
|  | 3 | 255 | 40.8 | 32.9-49.2 | 11.7 | 5.8-22.2 | 808 | 3.7 | 2.7-5.1 |
| woreda-level | | 841 | 30.9 | 24.0-38.9 | 8.0 | 4.9-12.6 | 2714 | 3.1 | 2.5-3.9 |
| **Woreillu** | 1 | 241 | 48.4 | 38.1-58.8 | 3.7 | 1.7-7.9 | 903 | 1.3 | 0.7-2.3 |
|  | 2 | 280 | 61.8 | 53.6-69.4 | 3.7 | 1.6-8.4 | 961 | 1.4 | 0.5-3.7 |
|  | 3 | 292 | 38.0 | 29.3-47.6 | 1.1 | 0.4-2.9 | 1039 | 0.6 | 0.3-1.4 |
| woreda-level | | 813 | 46.5 | 41.1-51.9 | 2.3 | 1.4-3.8 | 2903 | 0.9 | 0.6-1.6 |

*****estimates weighted according to selection probabilities adjusted for correlation in the data due to clustering at household, development team and gott level; CI, confidence interval; - no case identified
