# Supplementary material for: Prevalence of Trachoma at Sub-District Level in Ethiopia: Determining When to Stop Mass Azithromycin Distribution
Source: PLoS Negl Trop Dis. 2014 Mar 13;8(3):e2732. doi: 10.1371/journal.pntd.0002732 (PMC3953063; doi:10.1371/journal.pntd.0002732)
Supplement: Table S2 — Prevalence* of trachoma clinical signs by sub-district evaluation unit (EU) and woreda in South Gondar, Ethiopia, 2011. (DOCX) [file pntd.0002732.s003.docx]

**Table S2: Prevalence* of trachoma clinical signs by sub-district evaluation unit (EU) and *woreda* in South Gondar 2011**

| **South Gondar** | **EU** | **Children**  **1-9 years** | **TF** | | **TI** | | **All ages** | **TT** | |
| --- | --- | --- | --- | --- | --- | --- | --- | --- | --- |
| ***woredas*** |  |  | **%** | **95% CI** | **%** | **95% CI** |  | **%** | **95% CI** |
| **Debre Tabor** | 1 | 519 | 8.6 | 3.5-19.5 | 3.9 | 1.4-10.4 | 1109 | 1.0 | 0.0-2.2 |
| **Dera** | 1 | 606 | 31.0 | 17.6-48.5 | 6.9 | 4.7-9.8 | 1188 | 1.3 | 0.8-2.3 |
|  | 2 | 447 | 30.1 | 18.9-44.4 | 2.7 | 1.6-4.6 | 954 | 2.4 | 1.5-3.9 |
|  | 3 | 550 | 23.0 | 15.2-33.1 | 2.5 | 1.0-6.2 | 1133 | 1.0 | 0.4-2.3 |
|  | 4 | 524 | 26.1 | 15.1-41.1 | 5.5 | 2.8-10.9 | 1075 | 0.8 | 0.3-2.5 |
| woreda-level | | 2128 | 27.3 | 21.9-34.5 | 4.5 | 3.2-6.4 | 4350 | 1.3 | 0.9-1.9 |
| **East Estie** | 1 | 353 | 18.3 | 11.9-27.2 | 3.9 | 3.2-4.8 | 705 | 2.7 | 1.5-4.6 |
|  | 2 | 474 | 24.5 | 19.5-30.3 | 9.2 | 4.5-18.0 | 1008 | 1.7 | 0.8-3.6 |
|  | 3 | 486 | 14.5 | 10.0-20.4 | 5.6 | 3.5-9.0 | 1004 | 0.6 | 0.2-1.7 |
|  | 4 | 514 | 16.7 | 10.3-26.0 | 3.9 | 1.5-9.5 | 1144 | 1.1 | 0.8-1.5 |
| woreda-level | | 1828 | 18.9 | 16.1-22.2 | 6.1 | 4.1-8.9 | 3861 | 1.4 | 0.9-2.1 |
| **Ebinat** | 1 | 538 | 31.7 | 20.3-45.7 | 7.9 | 5.2-11.7 | 1173 | 1.9 | 1.1-3.1 |
|  | 2 | 446 | 44.8 | 35.0-55.1 | 12.7 | 9.2-17.3 | 991 | 3.4 | 1.9-5.9 |
|  | 3 | 506 | 55.7 | 47.7-63.4 | 13.4 | 7.1-23.7 | 971 | 1.9 | 0.9-4.0 |
|  | 4 | 537 | 43.4 | 35.3-51.9 | 8.4 | 3.1-20.6 | 1121 | 1.1 | 0.6-2.0 |
| woreda-level | | 2036 | 44.9 | 39.5-50.5 | 10.7 | 7.6-14.9 | 4256 | 2.0 | 1.4-2.7 |
| **Farta** | 1 | 458 | 22.3 | 16.2-28.4 | 6.0 | 3.3-10.6 | 962 | 2.0 | 1.0-4.0 |
|  | 2 | 516 | 10.6 | 7.2-13.9 | 1.4 | 0.8-2.5 | 1206 | 1.2 | 0.7-1.9 |
|  | 3 | 487 | 21.5 | 13.0-30.1 | 4.9 | 2.7-8.7 | 1159 | 2.4 | 1.5-3.7 |
|  | 4 | 446 | 22.2 | 17.8-26.6 | 7.1 | 4.4-11.2 | 921 | 2.1 | 1.1-4.0 |
| woreda-level | | 1907 | 17.8 | 14.2-21.4 | 4.3 | 3.1-5.8 | 4248 | 1.8 | 1.4-2.5 |
| **Fogera** | 1 | 437 | 28.9 | 23.3-35.3 | 6.0 | 4.0-8.7 | 1108 | 1.2 | 0.4-3.3 |
|  | 2 | 518 | 39.1 | 23.8-56.9 | 9.8 | 6.1-15.3 | 1227 | 2.2 | 1.3-3.6 |
|  | 3 | 444 | 28.7 | 22.6-35.8 | 8.1 | 4.0-15.8 | 1080 | 1.5 | 0.7-2.9 |
| woreda-level | | 1399 | 33.3 | 25.7-41.8 | 8.1 | 6.0-11.0 | 3415 | 1.7 | 1.0-2.7 |
| **Lay gayint** | 1 | 487 | 14.3 | 7.0-26.9 | 4.6 | 1.6-12.2 | 1142 | 1.1 | 0.6-2.1 |
|  | 2 | 512 | 15.3 | 11.3-20.5 | 7.8 | 5.6-10.8 | 1077 | 2.4 | 1.0-5.3 |
|  | 3 | 489 | 16.8 | 8.1-31.5 | 4.4 | 2.4-8.2 | 1246 | 1.6 | 0.9-2.8 |
|  | 4 | 481 | 30.0 | 17.7-46.0 | 8.4 | 5.0-14.0 | 1032 | 1.7 | 1.2-2.3 |
| woreda-level | | 1971 | 18.8 | 12.8-24.8 | 6.2 | 4.6-8.5 | 4497 | 1.6 | 1.2-2.4 |
| **Libokem** | 1 | 474 | 20.2 | 11.8-32.3 | 7.3 | 4.3-12.3 | 1048 | 1.2 | 0.6-2.6 |
|  | 2 | 496 | 19.1 | 12.9-27.6 | 6.0 | 3.1-11.3 | 1087 | 1.8 | 1.3-2.5 |
|  | 3 | 512 | 32.3 | 21.1-46.1 | 6.5 | 4.0-10.2 | 1154 | 1.3 | 0.7-2.2 |
| woreda-level | | 1482 | 24.3 | 18.1-31.9 | 6.5 | 4.7-9.0 | 3289 | 1.5 | 1.1-1.9 |
| **Simada** | 1 | 430 | 24.6 | 17.0-34.1 | 8.1 | 5.1-12.7 | 1043 | 4.2 | 2.6-6.6 |
|  | 2 | 503 | 25.0 | 19.7-31.1 | 10.3 | 6.6-15.9 | 1100 | 4.0 | 2.3-7.0 |
|  | 3 | 447 | 24.5 | 16.5-34.8 | 13.0 | 9.3-17.8 | 1115 | 2.2 | 1.0-4.7 |
|  | 4 | 416 | 21.3 | 12.8-33.4 | 2.7 | 1.4-5.1 | 877 | 4.5 | 3.0-6.4 |
| woreda-level | | 1800 | 23.9 | 19.8-28.5 | 8.8 | 6.9-11.2 | 4135 | 3.5 | 2.7-4.7 |
| **Tach gayint** | 1 | 455 | 36.5 | 23.9-51.2 | 10.5 | 6.2-17.2 | 1091 | 1.4 | 0.9-2.4 |
|  | 2 | 482 | 31.1 | 15.2-53.1 | 9.0 | 3.6-20.8 | 1094 | 2.7 | 1.4-5.1 |
| woreda-level | | 937 | 33.8 | 22.6-47.1 | 9.7 | 5.8-15.9 | 2185 | 2.0 | 1.3-3.3 |
| **West Estie** | 1 | 508 | 31.5 | 23.5-40.7 | 7.0 | 3.8-12.5 | 1169 | 1.1 | 0.7-1.7 |
|  | 2 | 501 | 29.7 | 18.7-43.7 | 11.8 | 7.0-19.7 | 1011 | 3.0 | 2.0-4.4 |
| woreda-level | | 1009 | 30.6 | 23.4-38.9 | 9.5 | 6.3-14.2 | 2180 | 2.0 | 1.5-2.7 |
| **Woreta town** | 1 | 543 | 17.2 | 9.7-28.7 | 6.7 | 3.6-12.1 | 1127 | 1.1 | 0.3-3.2 |

*****Estimates weighted according to selection probabilities adjusted for correlation in the data due to clustering at household, development team and *gott* level; CI, confidence interval
